# Comparative efficacy of antiangiogenic treatment for newly diagnosed glioblastoma: A protocol for systematic review and network meta-analysis

**DOI:** 10.1097/MD.0000000000020011

**Published:** 2020-05-08

**Authors:** Runting Li, Chao Li, Zhaolun Cai, Lianwang Li, Liudong Wei, Zenghui Qian, Dabiao Zhou

**Affiliations:** a Department of Neurosurgery, Beijing Tiantan Hospital, Capital Medical University, Beijing; b Department of Gastrointestinal Surgery, West China Hospital, Sichuan University, Chengdu 610041, Sichuan, China.

**Keywords:** antiangiogenic drugs, Bayesian network meta-analysis, glioblastoma, newly diagnosed glioblastoma, overall survival, progression-free survival, protocol

## Abstract

**Background:**

Glioblastoma is the most common malignant primary brain tumor which has highly expressed vascular endothelial growth factor. To date, various antiangiogenic drugs have been investigated in clinical trials but with no overall conclusion, especially for newly diagnosed glioblastoma (nGBM). In this study, Bayesian network meta-analysis will be used to conduct a comprehensive analysis of the results of different clinical trials, and assess the efficacy of different antiangiogenic drugs on nGBM.

**Methods:**

In order to find more comprehensive information about the application of antiangiogenic drugs in nGBM patients, we searched the MEDLINE, EMBASE, Web of Science, and Cochrane Central Register of Controlled Trials for relevant randomized controlled trials. We also reviewed their reference lists to avoid omissions. Cochrane risk of bias tool (V.1.4.3) and Stata (V.15.0) will be used to assess the methodological quality of this review.

**Results:**

This study will provide reliable evidence for different antiangiogenic therapies in nGBM patients.

**Conclusion:**

We will evaluate the relative effectiveness of different antiangiogenic drugs and rank each intervention in nGBM patients through prognosis to provide decision-making reference on which method to choose for clinicians.

**Protocol registration number:**

CRD42019146537

## 1 Introduction

Glioblastoma (GBM) is the most common malignant primary brain tumor, accounting for about 28% of all brain tumors and 80% of malignant brain tumors. GBM is also known for its invasive and aggressive behavior.^[1,2]^ Patients with newly diagnosed glioblastoma (nGBM) have a poor prognosis even when treated with maximal resection followed by radiotherapy combined with temozolomide (TMZ), as well as maintenance therapy with TMZ. The median survival time is 14 to 16 months, and tumor re-growth and patient relapse still remain inevitable.^[3–6]^ Moreover, once GBM recurs, the median overall survival (OS) time is typically 3 to 9 months, and available therapies have a limited impact on outcome.^[7]^

The biology of oncogenesis and the molecular mechanisms of GBM have showed that it typically overexpresses vascular endothelial growth factor, which can promote tumor angiogenesis, contributing to tumor growth and progression.^[8]^ Therefore, antiangiogenic therapy seems to be an attractive therapeutic strategy. Drawing on the experience of positive results from antiangiogenic therapy in other solid cancers, there have recently been a number of clinical trials of antiangiogenic drugs in GBM.^[9]^ Among those drugs, bevacizumab (BEV), a humanized monoclonal antibody against vascular endothelial growth factor, has already played a positive role when combined with standard therapy in recurrent diagnosed glioblastoma with both radiographic response and progression-free survival (PFS).^[10–13]^ In May 2009, the Food and Drug Administration approved BEV for the first-line treatment of recurrent diagnosed glioblastoma patients.^[14]^ Noteworthy, 2 studies in 2014 showed a longer PFS with BEV but failed to demonstrate an improvement in OS in nGBM.^[15,16]^ Trials of various other antiangiogenic drugs were conducted to assess the effectiveness in nGBM in the past few years, including dasatinib, temsirolimus, cilengitide, nimotuzumab, vandetanib, and everolimus, but the final results showed no significant difference in PFS or OS between antiangiogenic drug group and TMZ + radiotherapy group.^[17–22]^

To date, a number of traditional meta-analyses have been performed of the use of antiangiogenic drugs in GBM.^[23–29]^ However, traditional meta-analyses cannot provide integrated comparison of multiple interventions due to the lack of concurrent trials. Network meta-analysis (NMA) can help to solve this problem since it can compare all available treatments by pooling evidence from direct and indirect comparisons into 1 synthetic analysis. This can achieve a higher degree of precision in the estimation of the effectiveness of different interventions compared with traditional meta-analyses.^[30]^ In this protocol, we aim to conduct a NMA to compare the efficacy and safety of different antiangiogenic treatments for nGBM and to rank those treatment plans.

## 2 Methods

### 2.1 Protocol and registration

This NMA protocol was reported following the preferred reporting items for systematic review and meta-analysis protocols (PRISMA-P).^[31]^ Our protocol has been registered in the International Prospective Register of Systematic Review network. The International Prospective Register of Systematic Review registration number is CRD42019146537. The NMA will be conducted according to preferred reporting items for systematic review and meta-analysis extension vision statement (PRISMA-NMA).^[32]^

### 2.2 Ethics and dissemination

No ethical issues are foreseen. The results of present research will be published in a peer-reviewed journal.

### 2.3 Eligibility criteria

#### 2.3.1 Participants

The present study will include adult patients (>18 years) with newly diagnosed, histologically confirmed GBM.

#### 2.3.2 Interventions

We will include studies assessing the efficacy and safety of 2 or more of the following treatments: antiangiogenic drugs combined with standard chemoradiotherapy regimen, antiangiogenic drugs combined with cytotoxic drugs and standard chemoradiotherapy, or standard chemoradiotherapy regimen.

#### 2.3.3 Outcomes

The primary outcome is OS which is defined as the time between randomization and death from any cause.^[16]^ The secondary outcome is PFS which is defined as the time between randomization and either disease progression or death.^[33]^

#### 2.3.4 Study type

Only randomized controlled trials (RCTs) in English will be included in the present study. Meeting abstracts, letters, case reports, reviews, or nonclinical studies without usable data will be excluded.

### 2.4 Data source and search strategy

We systematically searched the PubMed, Embase (Ovid), and Cochrane Central Register of Controlled Trials for relevant RCTs until May 2019. The reference lists of included studies will be also checked for additional RCTs.^[34]^

Search strategy of PubMed was as follows:

#1 ((((((((((((((((((((((((((((bevacizumab) OR aflibercept) OR olaratumab) OR ramucirumab) OR cediranib) OR vatalanib) OR pazopanib) OR cabozantinib) OR sunitinib) OR sorafenib) OR vandetanib) OR AEE788) OR lenvatinib) OR tivozanib) OR enzastaurin) OR thalidomide) OR cilengitide) OR dasatinib) OR temsirolimus) OR lenalidomide) OR rofecoxib) OR ABT-510) OR regorafenib) OR apatinib) OR Trebananib) OR PF-04856884) OR AMG-780) OR nesvacumab) OR antiangiogenic

#2 ((((((((“Randomized Controlled Trial” [Publication Type]) OR “Controlled Clinical Trial” [Publication Type]) OR “randomized” [tiab]) OR “placebo” [tiab]) OR “Clinical Trials as Topic” [Mesh:NoExp]) OR “randomly” [tiab]) OR “trial” [ti])) NOT ((“Animals” [mh]) NOT “ humans” [mh])

#3 #1 AND #2

### 2.5 Selection of studies

Two authors (LRT and LC) will independently screen the titles and abstracts of all records after removing duplicates using EndNote Reference Manager Software (Clarivate Analytics, Philadelphia, PA). Only those meeting the eligibility criteria will be included. If studies have duplicate data, only the study with the most recent publication date and larger sample size will be chosen. The third author (CZL) will act as an arbitrator in the event of disagreement between the first 2 authors. The process of literature selection will be shown in a PRISMA flow diagram.^[35]^

### 2.6 Data extraction

The authors will extract following the data independently using a predefined spreadsheet: the name of the first author; year of publication; study duration; characteristics of interventions; follow-up time; sample size; age; and outcomes. We will contact corresponding authors of studies for answers to any questions that arise arisen during data extraction and for clarification of any areas of uncertainty in the methods and results.^[36]^ All data will be reviewed and separately extracted by 2 independent investigators (LRT and LC), and the third author (CZL) will act as an arbitrator.

### 2.7 Risk of bias assessment

The risk of bias in individual studies will be evaluated from 7 aspects (sequence generation, allocation concealment, blinding of participants, and personnel, incomplete outcome data, selective reporting, and other bias and risk), using the Cochrane Collaboration tool.^[37,38]^ Each item will be evaluated at 3 levels: low risk, unclear, and high risk. Two authors (LRT and LC) will conduct quality assessment independently and any disagreement will be solved by discussion with the third author (CZL).

### 2.8 Geometry of the network

Stata 12 (Stata Corp, College Station, TX) will be used to draw network plots to depict the available evidence. In the network plot, the size of nodes represents the number of studies evaluating each treatment, and the thickness of the lines between the nodes represent the number of direct comparisons between tests.^[34,39,40]^

### 2.9 Data synthesis and statistical methods

Time-to-event outcomes will be assessed by calculating hazard ratios. Dichotomous outcomes will be analyzed by calculating the relative risks. Results from the NMA will be presented as summary relative effect sizes (hazard ratios or relative risks) and relative 95% confidence intervals for each possible pair of treatments.

We will first conduct a standard pairwise meta-analysis of all the direct comparisons with Stata (Stata Corp), using a random-effects model. Heterogeneity variances for each pairwise comparison will be estimated by *Q*-test and *I*^2^ statistic.^[41]^

Next, we will perform the NMA using R x64 3.5.0 and Stata (StataCorp). The inconsistency of our results will be confirmed by the node-splitting method and its Bayesian *P*-value.^[42]^ We will estimate the potential ranking probability of interventions by calculating the surface under the cumulative ranking curve (SUCRA) for each intervention.^[43]^ The SUCRA value ranges between 0 and 1, and the intervention with a higher SUCRA value is considered to have better efficacy.^[40]^

Subgroup analysis will be performed based on O-6-methylguanine–DNA methyltransferase (MGMT) status and recursive partitioning analysis (RPA) class.

We will use comparison-adjusted funnel plots to evaluate the small study effects in the present study.^[44]^

## 3 Discussion

This will be the first NMA to comprehensively compare the efficacy of different antiangiogenic drugs in nGBM patients. Despite the advantages of this approach, there are some inevitable limitations. Some antiangiogenic drugs are not discussed in the literature due to the lack of RCTs or the RCT is still ongoing. The potentially high heterogeneity among different studies may also influence the final results of this NMA. However, we hope this study will uncover the best antiangiogenic treatment currently available for clinical practice and assist in directing future study design.

## Author contributions

**Conceptualization:** Dabiao Zhou, Chao Li.

**Data curation:** Zhaolun Cai.

**Investigation:** Runting Li, Zenghui Qian.

**Methodology:** Zhaolun Cai.

**Project administration:** Dabiao Zhou, Runting Li.

**Resources:** Dabiao Zhou, Runting Li.

**Software:** Zhaolun Cai.

**Supervision:** Dabiao Zhou.

**Validation:** Zhaolun Cai.

**Writing – original draft:** Runting Li, Chao Li.

**Writing – review and editing:** Dabiao Zhou, Zhaolun Cai.
